# Multiple and large simple renal cysts are associated with glomerular filtration rate decline: a cross-sectional study of Chinese population

**DOI:** 10.1186/s40001-023-01552-2

**Published:** 2024-01-03

**Authors:** Cheng Jin, Lu Wei, Zhenzhu Yong, Yao Ma, Bei Zhu, Xiaohua Pei, Canhong Zhu, Weihong Zhao

**Affiliations:** 1 Division of Nephrology, Department of Geriatric, The First Affiliated Hospital of Nanjing Medical University, No. 300 Guangzhou Road, Nanjing, 210029 Jiangsu China; 2 Department of Geriatrics, The Affiliated People’s Hospital of Jiangsu University, Zhenjiang, 212002 Jiangsu China

**Keywords:** Simple renal cyst, Glomerular filtration rate, Kidney function

## Abstract

**Background:**

Although simple renal cyst (SRC) is a kind of structural alterations of kidney with age, the relationship between SRC and renal function is still obscure. We investigated the relationship between SRC and renal function in Chinese population.

**Methods:**

The medical records of 41,842 individuals who underwent physical examinations at the Health Check-up Center at our institution in 2018 were reviewed. According to whether with SRC, they were divided into no-SRC and SRC groups. SRCs were classified into subgroups based on number (< 2 vs. ≥ 2) and size (< 2 cm vs. ≥ 2 cm). Logistic regression was used to examine the relationship between SRC and estimated glomerular filtration rate (eGFR).

**Results:**

Multinomial logistic regression analysis showed that the adjusted odds ratio (OR) for eGFR slight decline in subjects with SRC was 1.26(95% confidence interval (95% CI):1.17–1.35, *p* < 0.001), and the OR for eGFR severe decline was 1.35(95% CI: 1.16–1.56, *p* < 0.001) compared with no-SRC. The adjusted OR of SRC number ≥ 2 and ≥ 2 cm on the risk of eGFR severe decline was the highest (OR:1.68, 95% CI:1.25–2.23, *p* < 0.01) of four SRC subgroups.

**Conclusions:**

SRC is related to eGFR decline, especially when the person with one more SRCs and the size of SRC is more than 2 cm. SRC could be a warning sign for clinicians to judge the decline of renal function.

**Supplementary Information:**

The online version contains supplementary material available at 10.1186/s40001-023-01552-2.

## Background

The kidney is one of the most susceptible organs to aging [1, 2]. Structural and functional alterations occur in the kidney with age. Kidney function declines with age is mainly manifested as a decline in glomerular filtration rate (GFR) [3, 4]. Renal cyst is one of the structural alterations of kidney with age. According to the malignant tendency, renal cysts are categorized into four Bosniak classes baes on ultrasound, computed tomography (CT) and/or magnetic resonance imaging (MRI). Bosniak I renal cysts are the most common and are usually called simple renal cysts (SRCs) [5, 6]. On ultrasonic images, SRC is characterized by homogeneous cystic cavity, regular contour, clear boundary, thin smooth wall, no echo, and no septa or calcifications [5]. In the past, it was considered that SRC had no obvious clinical symptoms, low malignant tendency, little impact on body function, and no follow-up was required [5, 6]. Recently, more and more researchers believe that SRC is not as simple as previously thought [7–10]. Clinical studies found that the prevalence of SRC increased with age [11]. In the age-related comorbidity population, such as diabetes, arteriosclerosis, hyperuricemia and hypertension, the SRC prevalence is high [7, 8, 12–14].

The relationship between SRC and renal function is still controversial [15–18]. Some scholars such as HJ Chin [19], Ozdemir [18] believe that SRC has nothing to do with the decline of renal function, while other scholars such as Kong X [15], AL-Said J [17] believe that SRC is related to the decline of renal function. Therefore, this study intends to explore the relationship between SRC and renal function in Chinese population through a cross-sectional study.

## Methods

### Study population

**Fig. 1:**
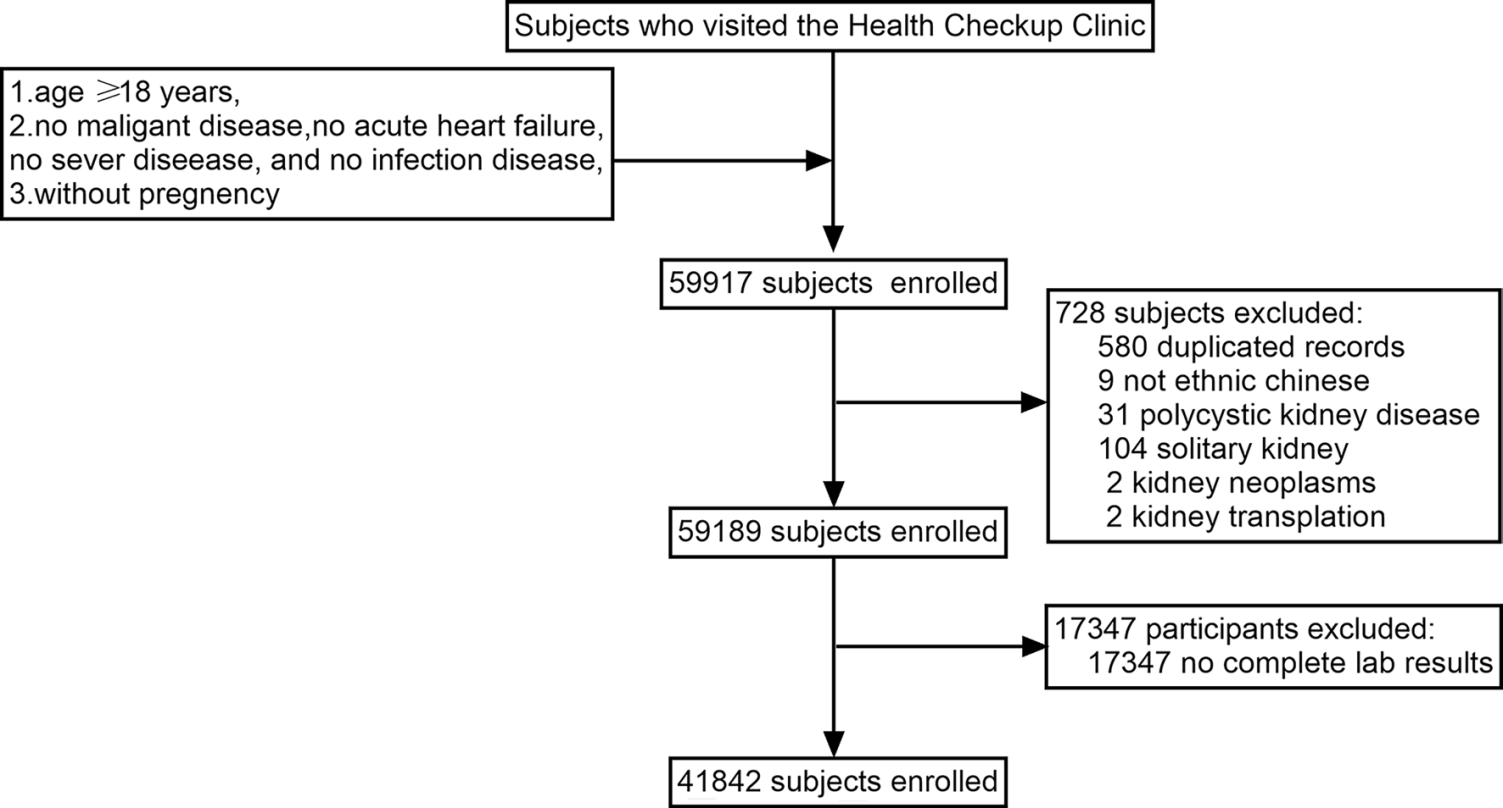
Flowchart of the enrolled subjects

The subjects of this cross-sectional study were individuals who underwent physical examinations from January to December 2018 at the Health Check-up Center of The Affiliated People’s Hospital of Jiangsu University, which is a tertiary teaching hospital in China. A total of 41,842 participants were collected for this study and fulfilled the following criteria:

Inclusion criteria: (1) age ≥ 18 years; (2) no malignant disease, no acute heart failure, no sever disease or infection disease;(3) without pregnancy. Exclusion criteria: (1) not Chinese ethnic origin, (2) a history of renal transplantation; (2)images of polycystic kidney disease(PKD), solitary kidney, partial nephrectomy, renal tumor, or ectopic kidney; (3) missing data for other variables. For subjects with multiple physical examinations, only the first exam record was used. The flowchart of subject inclusion is summarized in Fig. 1.

This study was reviewed and approved by the Ethics Committee of The Affiliated People’s Hospital of Jiangsu University (ZFPH No: K-20220061-W). Given the retrospective study design, the need for formal consent was waived. The authors complied strictly with the Declaration of Helsinki and covered patient data confidentiality.

### Clinical variables

Body mass index (BMI) was calculated based on body weight (kilograms)/height(meters) squared. All data were obtained retrospectively via the hospital electronic database. Basic demographic characteristics, height, weight, systolic blood pressure, diastolic blood pressure, fasting plasma glucose (FPG), glycosylated hemoglobin (HbA1c), total cholesterol (TC), triglyceride (TG), high-density lipoprotein cholesterol (HDL-C), serum creatinine and other data were recorded.

Hypertension was defined as systolic blood pressure ≥ 140 mmHg or diastolic blood pressure ≥ 90 mmHg or with a positive history of hypertension. Mean blood pressure = (systolic blood pressure_+_ diastolic blood pressure × 2)/3.

Diabetes mellitus was defined as FPG ≥ 7.0 mmol/L or HbA1c > 6.5% or with a positive history of diabetes mellitus.

### SRC diagnosis

Ultrasonic diagnosis: According to Bosniak method, SRC was diagnosed on the basis that: (1) round or oval cystic cavity; (2) regular outline of the cystic cavity and clear boundary; (3) homogeneous cystic cavity without separation or calcification;(4) thin and smooth wall without echo, and (5) enhanced echo of the posterior wall [5].

CT diagnosis: CT images showed uniform water attenuation (range -10hu to 20hu), thin and smooth wall, no calcification, septum or mural nodules [5, 20]. If the subject not only had kidney ultrasound record, but also had abdominal CT record, the CT record were prevailed. The subjects with SRC were further stratified according to the maximum diameter of SRC (< 2 cm vs. ≥ 2 cm) and the number of SRC (< 2 vs. ≥ 2).

### eGFR calculation

The estimated glomerular filtration rate (eGFR) was calculated by Chronic Kidney Disease Epidemiology Collaboration (CKD-EPI) creatinine Eq. (2009) [21]. The equation is as follows:

for females,

if SCr ≤ 0.7 mg/dl, eGFR = 144 × (SCr/0.7) ^−0.329^ × (0.993) ^age^,

and if SCr > 0.7 mg/dl, eGFR = 144 × (SCr/0.7) ^−1.209^ × (0.993) ^age^;

for males,

if SCr ≤ 0.9 mg/dl, eGFR = 141 × (SCr/0.9) ^−0.411^ × (0.993) ^age^,

and if SCr > 0.9 mg/dl, eGFR = 141 × (SCr/0.9) ^−1.209^ × (0.993) ^age^,where Scr is serum creatinine (mg/dl).

### Statistical analysis

All subjects were divided into no simple renal cyst group (no-SRC group) and simple renal cyst group (SRC group) according to whether they had simple renal cyst. The SRC group was further divided into subgroups based on the number and size of cysts (SRC number < 2 and size < 2 cm, SRC number < 2 and size ≥ 2 cm, SRC number ≥ 2 and size < 2 cm and SRC number ≥ 2 and size ≥ 2 cm). Categorical variables were presented as counts (percentages). Continuous variables were described as mean ± standard deviation or the median (interquartile range) according to normal or skewed distribution. Student’s t-test was used to compare between two groups of normally distributed data, and the Mann–Whitney U test was used for skewed distributions. One-way analysis of variance (ANOVA) test was used to analyze the significance among multi-groups. The counting data were tested using Chi-square test. Logistic regression was employed to identify associations between SRC prevalence and eGFR. Using IBM ® SPSS ® Statistical version 23.0 (IBM, New York, USA) for statistical analysis. Two tailed* p* value < 0.05 was considered statistically significant. Prism 9.0 (Graphpad, San Diego, CA, USA) was used for plotting graphs.

## Results

### Baseline characteristics

According to the inclusion and exclusion criteria, 41,842 subjects were finally included, as shown in Fig. 1. Among them, there were 5758 subjects in the simple renal cyst group (SRC group) and 36,084 subjects in the no simple renal cyst group (no-SRC group). The age of these subjects ranged from 18 to 101 years old with the average of 50.6 ± 14.8 years old. Baseline characteristics are shown in Table 1. Compared to no-SRC group, subjects in SRC group were older, and with higher proportion of males, higher systolic blood pressure, higher diastolic blood pressure, higher mean arterial pressure, higher HbA1c, higher FPG, higher TG, higher urea nitrogen, higher serum creatinine, higher uric acid, but lower HDL-C and lower eGFR (*p* < 0.05).

**Table 1:** Characteristics of the study subjects

Variables	Total (*N* = 41,842)	no-SRC (*N* = 36,084)	SRC (*N* = 5758)	F or *χ*^2^	*P* value
Male gender	25,971 (62.1)	21,784 (60.4)	4187 (72.7)	321.482	< 0.001
Age(years)	50.6 ± 14.8	49.7 ± 14.6	56.7 ± 14.7	1.896	< 0.001
≥ 60 years	10,531 (25.2)	8338 (23.1)	2193 (38.1)	591.555	< 0.001
Body mass index(kg/m2)	22.6 ± 4.7	22.7 ± 4.7	22.6 ± 4.6	7.871	0.256
Systolic blood pressure (mmHg)	125.9 ± 18.8	125.3 ± 18.6	130 ± 19.5	27.181	< 0.001
Diastolic blood pressure(mmHg)	76.8 ± 11.6	76.6 ± 11.6	78.5 ± 11.7	0.95	< 0.001
Mean arterial pressure (mmHg)	93.2 ± 13	92.8 ± 13	95.7 ± 13.1	1.00	< 0.001
Hypertension	10,720 (25.6)	8827 (24.5)	1893 (32.9)	184.461	< 0.001
HbA1c (%)	6.0 ± 1.0	5.9 ± 0.9	6.1 ± 1.1	11.54	< 0.001
Albumin(g/L)	44.7 ± 2.5	44.8 ± 2.5	44.4 ± 2.5	0.105	< 0.001
Fasting plasma glucose(mmol/L)	5.4 ± 1.4	5.3 ± 1.4	5.6 ± 1.6	114.133	< 0.001
Diabetes mellitus	2953 (7.0)	2359 (6.5)	576 (10.0)	91.455	< 0.001
HDL-cholesterol (mmol/L)	1.4 ± 0.3	1.4 ± 0.3	1.3 ± 0.3	0.055	< 0.001
LDL- cholesterol (mmol/L)	2.8 ± 0.7	2.8 ± 0.7	2.8 ± 0.7	2.202	0.524
Thyroid stimulating hormone (μIU/ml)	2.4 ± 2.1	2.4 ± 2	2.3 ± 2.5	0.095	0.305
Free triiodothyronine (pmol/L)	5.4 ± 0.7	5.4 ± 0.8	5.4 ± 0.7	1.975	0.105
Free thyroxine (pmol/L)	10.7 ± 1.9	10.7 ± 1.9	10.7 ± 1.7	1.222	0.625
Triglyceride(mmol/L)	1.68 ± 1.47	1.67 ± 1.48	1.74 ± 1.40	0.005	0.001
Total cholesterol (mmol/L)	4.8 ± 0.9	4.8 ± 0.9	4.8 ± 0.9	1.395	0.162
Urea nitrogen (mmol/L)	5.0 ± 1.4	5.0 ± 1.4	5.2 ± 1.5	18.938	< 0.001
Serum creatinine(mg/dl)	0.80 ± 0.25	0.80 ± 0.25	0.83 ± 0.22	0.003	< 0.001
eGFR[ml min^−1^ (1.73m^2^)^−1^]	99.1 ± 17.3	99.9 ± 17.2	94.0 ± 17.2	0.181	< 0.001
< 60	1376 (3.3)	1115 (3.1)	261 (4.5)		
60– < 90	9143 (21.8)	7377 (20.4)	1766 (30.7)		
≥ 90	31,323 (74.9)	27,592 (76.5)	3731 (64.8)		
Uric acid(μmol/L)	335.6 ± 87.9	334.4 ± 88.2	343.3 ± 85.9	6.474	< 0.001

Data are expressed as mean ± standard deviation or number (percentage)

SRC, simple renal cyst; HDL, high-density lipoprotein, LDL, low-density lipoprotein; HbA1c, glycosylated hemoglobin; eGFR, estimated glomerular filtration rate calculated by the 2009 CKD-EPI formula

### SRC and age

**Fig. 2:**
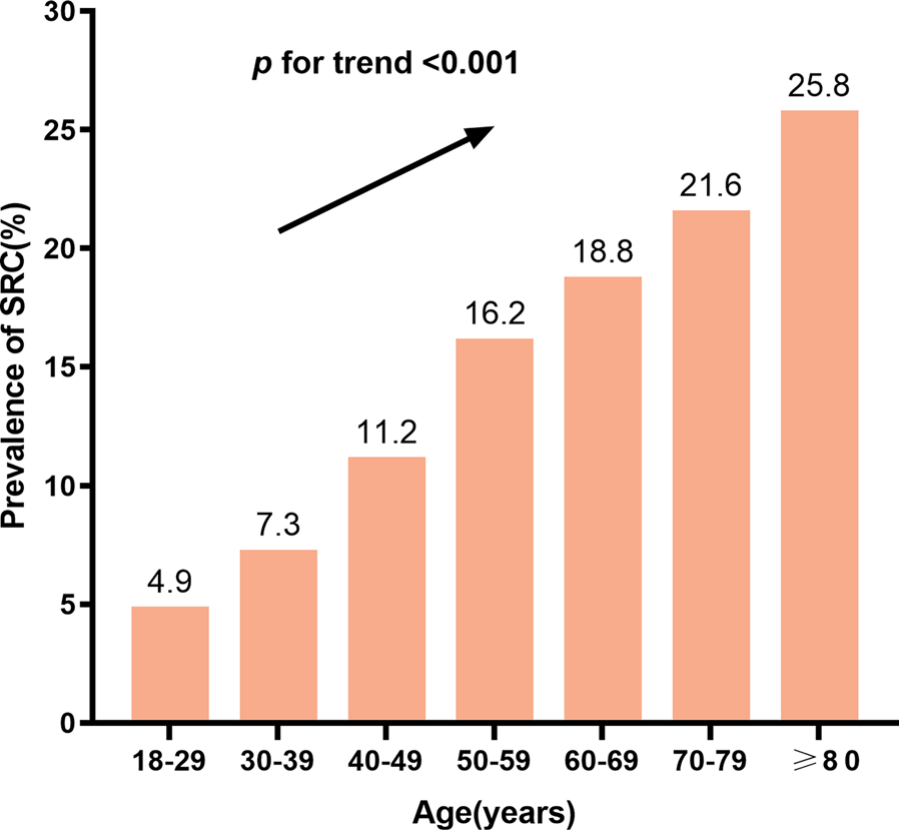
The prevalence of simple renal cyst (SRC) stratified according to age

**Fig. 3:**
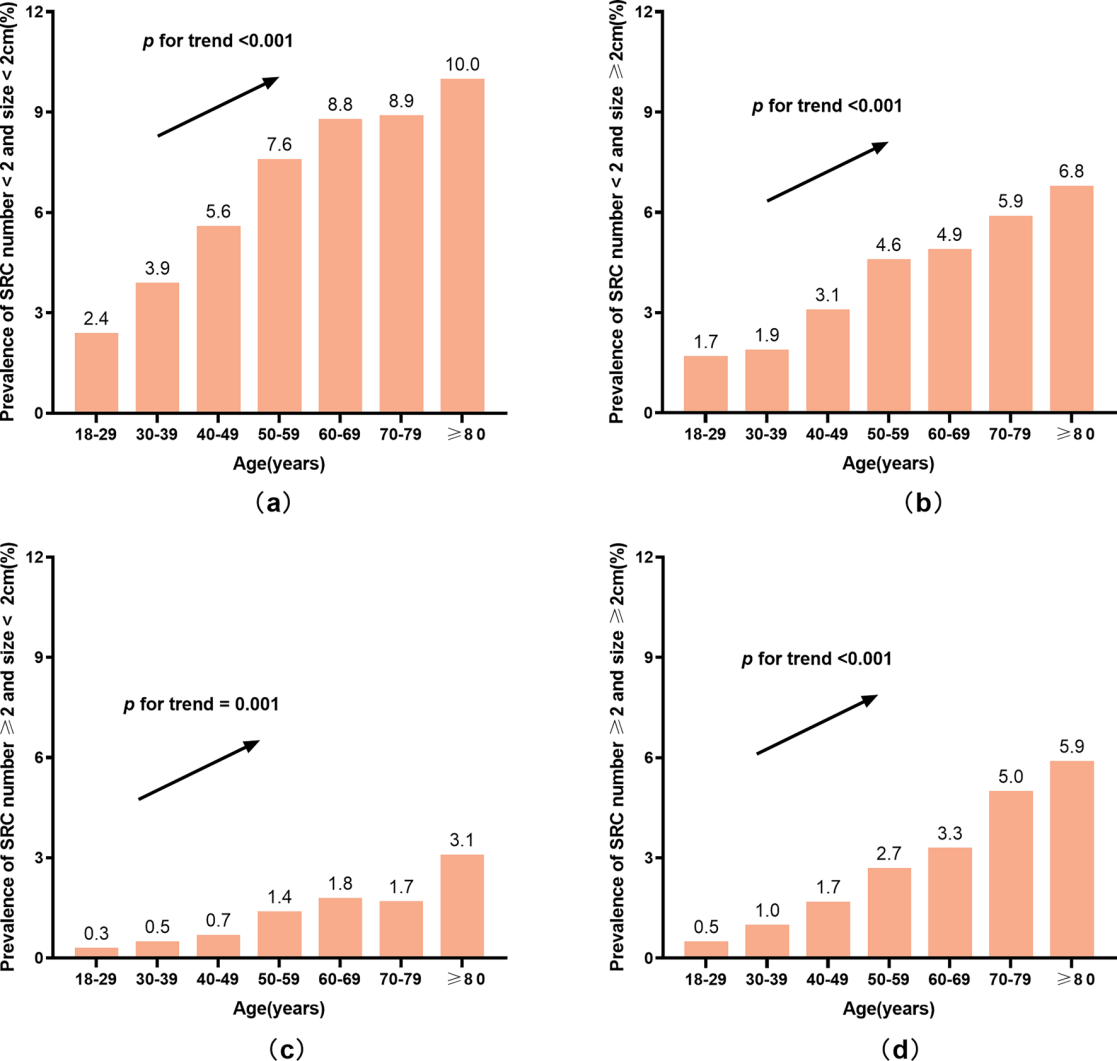
The prevalence of simple renal cyst (SRC) combining both cyst number and size. **a** Prevalence of SRC number < 2 and size < 2 cm. **b** Prevalence of SRC number < 2 and size ≥ 2 cm. **c** Prevalence of SRC number ≥ 2 and size < 2 cm. **d** Prevalence of SRC number ≥ 2 and size ≥ 2 cm

In accordance with their ages, subjects were stratified 7 layers: 18–29 years old, 30–39 years old, 40–49 years old, 50–59 years old, 60–69 years old, 70–79 years old, 80 years old and above. The prevalence of SRC in each layer was 4.9%, 7. 3%, 11. 2%, 16. 2%, 18. 8%, 21. 6% and 25.8%, respectively, showing an increasing trend (*p* < 0.001) (Fig. 2). When combining both cyst number and size in the analysis, the increasing trend stood still (Fig. 3).

### SRC and eGFR

**Fig. 4:**
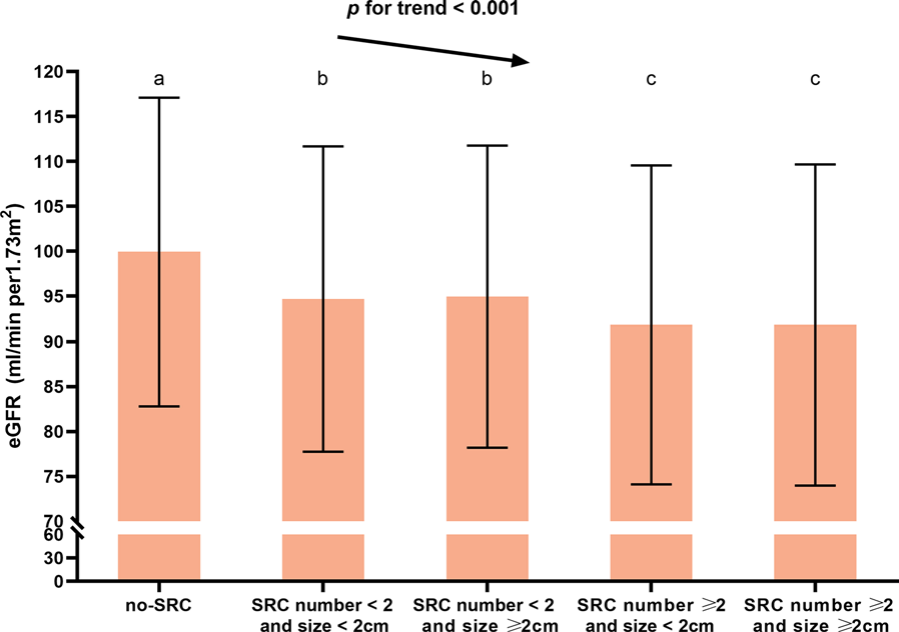
Comparisons of eGFR among subjects with simple renal cyst (SRC) and those without SRC (no-SRC). Error bars indicate standard errors. The same letter on the column indicates that the difference is not significant (*p* ≥ 0.05), and different letters **a**–**c** indicate that the difference is significant (*p* < 0.05). *eGFR* estimated glomerular filtration rate calculated by the 2009 CKD-EPI formula

As shown in Fig. 4, eGFR of no-SRC, SRC number < 2 and size < 2 cm, SRC number < 2 and size ≥ 2 cm, SRC number ≥ 2 and size < 2 cm and SRC number ≥ 2 and size ≥ 2 cm were 99.94 ± 17.16 ml/min per 1.73m^2^, 94.71 ± 16.97 ml/min per 1.73m^2^, 94.97 ± 16.78 ml/min per 1.73m^2^ and 91.83 ± 17.72 91.82 ± 17.85 ml/min per 1.73m^2^, showing a decreasing trend (*p* for trend < 0.001), and the eGFR of SRC number ≥ 2 and size ≥ 2 cm was the lowest of all. eGFR of the two SRC number ≥ 2 groups were lower than those of SRC number < 2 groups(*p* < 0.05). However, there were no statistically significant differences between the two SRC number ≥ 2 groups (*p* ≥ 0.05). And there were no statistically significant differences between the two SRC number < 2 either (*p* ≥ 0.05).

### Association between SRC and eGFR

A negative association was displayed between the prevalence of SRC and eGFR (β = -0.430, *p* < 0.05) after adjusted for gender, age (per 10 years), systolic blood pressure, fast glucose, serum uric acid and body mass index. In elderly population (age ≥ 60 years old), this kind of association was not statically significant (*β* = − 0.102, *p* > 0.05) after adjusted for clinical influencing factors, even though which was appeared before adjusted (*β* = − 1.173, *p* > 0.01). The negative association (*β* = − 0.660, *p* < 0.01) between the prevalence of SRC and eGFR was appeared still in adults (age < 60 years old) after adjusted. It could be noted that the prevalence of SRC showed stronger negative correlation with eGFR in younger adults than in the older adults (Table 2).

**Table 2:** Association between the prevalence of SRC and eGFR ^a^

	The adults		The elderly	
	*β*	*P* value	*β*	*P* value
With SRC	− 0.660	0.003	− 0.102	0.728

*SRC* simple renal cyst, *eGFR* estimated glomerular filtration rate calculated by the 2009 CKD-EPI formula

^a^Adjusted for gender, age, systolic blood pressure, fast glucose, serum uric acid and body mass index

### Association between SRC and eGFR decline

The association between eGFR decline and SRC was further analyzed. When eGFR slight decline (60 ≤ eGFR < 90 ml/min per1.73m^2^) and severe decline (eGFR < 60 ml/min per 1.73m^2^) were taken as dependent variables, the parallel line test failed (*p* < 0.05). Therefore, subsequent studies on the above dependent variables were conducted by multinomial logistic regression analysis instead of ordinal logistic regression analysis (Additional file 1: Table S1).

**Fig. 5:**
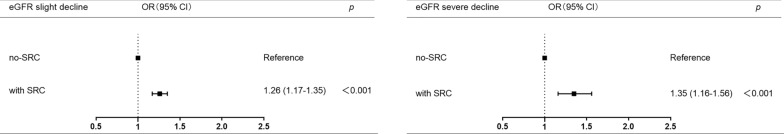
The association of eGFR decline and SRC. Adjusted by age, gender, hypertension, fasting plasma glucose, uric acid, and triglyceride. eGFR slight decline,60 ≤ eGFR < 90 ml/min per1.73m^2^; eGFR severe decline, eGFR < 60 ml/min per1.73m^2^. *OR* odds ratio, *CI* confidence interval, *eGFR* estimated glomerular filtration rate calculated by the 2009 CKD-EPI formula, *SRC* simple renal cyst

The presence of SRC was independently associated with eGFR slight decline and severe decline after adjusting for some clinical variables such as older age, gender, hypertension, fasting plasma glucose, uric acid, and triglyceride. Compared with no-SRC, the adjusted odds ratio (OR) for eGFR slight decline in subjects with SRC was 1.26 (95% confidence interval (95%CI): 1.17–1.35, *p* < 0.001), and the OR for eGFR severe decline was 1.35 (95%CI: 1.16–1.56, *p* < 0.001), increased by 9% than which of eGFR slight decline (Fig. 5).

**Fig. 6:**
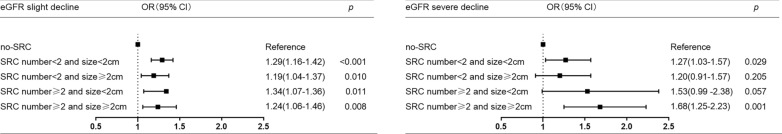
The association of eGFR decline and SRC four combinates. Adjusted by age, gender, hypertension, fasting plasma glucose, uric acid, and triglyceride. eGFR slight decline, 60 ≤ eGFR < 90 ml/min per1.73m^2^; eGFR severe decline, eGFR < 60 ml/min per1.73m^2^. *OR* odds ratio, *CI* confidence interval, *eGFR* estimated glomerular filtration rate calculated by the 2009 CKD-EPI formula, *SRC* simple renal cyst

**Fig. 7:**
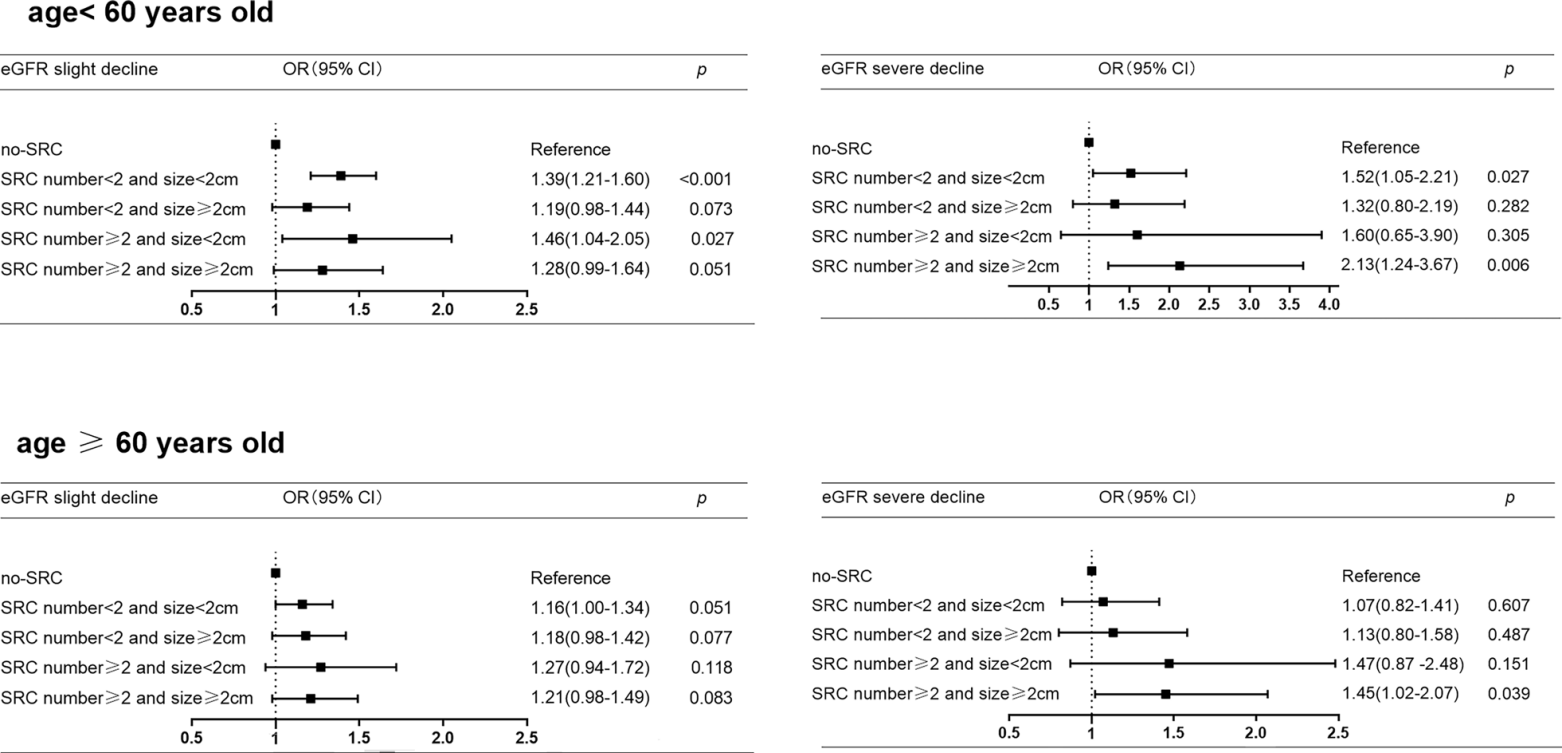
The association of eGFR decline and SRC four combinates stratified by age. Adjusted by gender, hypertension, fasting plasma glucose, uric acid, and triglyceride. eGFR slight decline, 60 ≤ eGFR < 90 ml/min per1.73m^2^; eGFR severe decline, eGFR < 60 ml/min per1.73m^2^; *OR* odds ratio, *CI* confidence interval; *eGFR* estimated glomerular filtration rate calculated by the 2009 CKD-EPI formula, *SRC* simple renal cyst

After adjusting for clinical variables such as age, gender, hypertension, fasting plasma glucose, uric acid, and triglyceride, the adjusted OR of SRC number ≥ 2 and ≥ 2 cm on the risk of eGFR severe decline was the highest of all (OR:1.68, 95% CI:1.25–2.23, *p* < 0.01) (Fig. 6). The association did not change after stratified by age. The adjusted OR of SRC number ≥ 2 and ≥ 2 cm on the risk of eGFR severe decline was 2.13(CI:1.24–3.67, *p* < 0.01) in adults (age < 60 years old), and 1.45(CI:1.02–2.07, *p* < 0.05) in older (age ≥ 60 years old) (Fig. 7).

## Discussion

SRCs are common renal cysts and commonly asymptomatic. With the advances in imaging technology, more and more SRCs are incidentally found during imaging examination for other reasons [6]. Due to different races, different complications, different imaging methods used, and other factors, the prevalence of SRC varies between 4.2% to 55% [22–24]. The detection rate of SRC by CT or MRI is higher than that by ultrasound [25]. The prevalence of SRC in the physical examination population is 7.7–10.5% [15, 26], and the prevalence of SRC in individuals with diabetes mellitus, hypertension is 21.1% [27] and 20.3% [28], respectively. The prevalence of SRC increases with age, and is reported to be up to 31.9% in the elderly [12]. The prevalence of SRC in this study was 13.8%, slightly higher than the study carried out by Chinese scholar Kong X et al. [15]. The reason is possibly caused by the fact that our study included CT examination results, which improved the detection rate. Our results showed the prevalence of SRC increased with age, which was consistent with previous studies. Concerning SRC number and size, the increasing trend with age stood still.

Some scholars hold the view that SRC could be a variant of "normal" [29], which has no special significance in clinical diagnosis or treatment and does not need follow-up [6, 30]. However, other scholars believe that SRC could be a kind of variant “without known pathologic associations” but for “with unknown pathologic associations” [31]. And its clinical significance still needs to be explored [12, 24, 32, 33]. SRC, simple renal cyst may not be as simple as we think. As early as 1977, a study by Baert L et al. [34] pointed out that before the identification of SRC by imaging, there had been microstructural changes in the kidney, including the formation of diverticula and micro-cysts. His study found that all kidneys of adults and elderly people had diverticula on the distal tubule, the number increasing with age. This change is consistent with our result that the prevalence of SRC increases with age.

SRC is not only related to aging, but also related to hypertension [8, 28], diabetes [12], arteriosclerosis[7] and hyperuricemia [13].In this study, we also found that the blood pressure, fasting plasma glucose, blood lipid, uric acid and serum creatinine in SRC group were higher than those in no-SRC group (*p* < 0.001), and eGFR in SRC group was lower than those in no-SRC group (*p* < 0.001).

The relationship between SRC and renal function is still controversial [15–18]. Some studies consider that SRC has nothing to do with the reduced renal function [18, 19], while others suggest that SRC is related to the decline of renal function [15, 17]. In this study, the presence of SRC was independently associated with eGFR slight decline and severe decline after adjustment. Analyzing SRCs with characteristics of both number and size, four SRC subgroups were all independently associated with eGFR slight decline and severe decline when other clinical variables were not adjusted (Additional file 2: Fig. S1). After adjusting for clinical variables, all four SRC subgroups were associated with eGFR slight decline, but were not all statistically associated with eGFR severe decline. The association of SRC number < 2 and size ≥ 2 cm and eGFR severe decline was not statistically significant. Except for "single large cyst", the OR on risk of eGFR severe decline was gained with the increase of SRC number and diameter. Among four SRC subgroups, the adjusted OR of SRC number ≥ 2 and ≥ 2 cm on the risk of eGFR severe decline was the highest of all (OR:1.68, 95% CI:1.25–2.23, *p* < 0.01). The association did not change after stratified by age, even though it was attenuated in older layer.

The following factors may explain the association between SRC and renal function decline. The association between SRC and renal function may be related to the aging process and primary renal diseases, which are accompanied by an increased number and enlarged size of cysts. SRC originated from distal convoluted tubule or collecting duct, and its precursor structure, renal tubular diverticulum or microcyst, is considered to be secondary to nephron loss [34]. With the advancing age and the occurrence of kidney diseases, the mass of renal cortex decreases, and nephrons losing increase the workload of the remaining nephrons, and may lead to hypertrophy or proliferation of renal tubular cells, leading to the formation of cysts [11]. On the other hand, some studies have shown the prevalence of SRC is related to the increased renin release which due to renal ischemia caused by cyst expansion [28, 35]. With the stimulation of renin angiotensin aldosterone system, the elevated blood pressure leads to endothelial dysfunction and microvascular ischemia, glomerulosclerosis, loss of nephron, and decline of renal function, which in turn causes the occurrence of renal cyst [36, 37]. The presence of SRC and the increase of SRC number or larger diameter will aggravate renal ischemia, because the effect of cyst expansion is more serious. Therefore, it is more likely to generate higher internal hydrostatic pressure to compress the surrounding renal tissue, leading to renal ischemia.

To sum up, this study believes that SRC has a certain correlation with the decline of eGFR. The greater the number and diameter of SRC, the lower the eGFR may be. This study has some limitations. 1.This study is a cross-sectional study that limits causal inference: we see correlations, but we cannot confirm the direction or causal relationship of these correlations. 2. Although this study included some abdominal CT results, the imaging basis of most subjects was ultrasound. Although renal ultrasound is a reliable tool for estimating renal length, it is not as sensitive as CT or MRI, so some smaller cysts may be missed in the study.3. The subjects of the study are ethnic Chinese. Whether the results of this study are applicable to other ethnic groups needs further research. 4. In this study, molecular genetic tests were not performed on every subject, especially the SRC group, to exclude PKD. Some people in our study may have PKD. In the follow-up, further studies were conducted to explore the causal relationship between SRC and eGFR decline.

## Conclusions

In conclusion, we believe that SRC is related to the decrease of eGFR, especially when the person with one more SRCs and the diameter of the biggest SRC is more than 2 cm. SRC may be a warning sign for clinicians to judge the decline of renal function.

### Supplementary Information

**Additional file 1: Table S1.** The adjusted OR of clinical variables on the risk of eGFR decline on the basis of multinomial logistic regression (OR, 95% CI).

**Additional file 2: Figure S1.** The unadjusted OR of SRC four combinates on the risk of eGFR decline. eGFR slight decline, 60 ≤ eGFR < 90 ml/min per1.73m^2^; eGFR severe decline, eGFR < 60 ml/min per1.73m^2^; *OR* odds ratio, *CI* confidence interval, *eGFR* estimated glomerular filtration rate calculated by the 2009 CKD-EPI formula, *SRC* simple renal cyst.

## Data Availability

The datasets used and analyzed during the current study are available from the corresponding author on reasonable request.

## Abbreviations

SRC: Simple renal cyst
OR: Odds ratio
CI: Confidence interval
eGFR: Estimated glomerular filtration rate
CT: Computed tomography
MRI: Magnetic resonance imaging
PKD: Polycystic kidney disease
BMI: Body mass index
FPG: Fasting plasma glucose
HbA1c: Glycosylated hemoglobin
TC: Total cholesterol
TG: Triglyceride
HDL-C: High-density lipoprotein cholesterol
CKD-EPI: Chronic Kidney Disease Epidemiology Collaboration
ANOVA: One-way analysis of variance
