# Supplementary material for: Multiple and large simple renal cysts are associated with glomerular filtration rate decline: a cross-sectional study of Chinese population
Source: Eur J Med Res. 2024 Jan 3;29:11. doi: 10.1186/s40001-023-01552-2 (PMC10763358; doi:10.1186/s40001-023-01552-2)
Supplement: Supplementary file 1 — Additional file 1: Table S1. The adjusted OR of clinical variables on the risk of eGFR decline on the basis of multinomial logistic regression (OR, 95% CI). [file 40001_2023_1552_MOESM1_ESM.docx]

**Additional file 1: Table S1.** The adjusted OR of clinical variables on the risk of eGFR decline on the basis of multinomial logistic regression (OR, 95% CI)

|  | **Model 1** | | |  | **Model 2** | | |
| --- | --- | --- | --- | --- | --- | --- | --- |
| **Variables** | **60≤eGFR＜90 vs. eGFR≥90**  **OR(95% CI)** |  | **eGFR＜60 vs. eGFR≥90**  **OR(95% CI)** |  | **60≤eGFR＜90 vs. eGFR≥90**  **OR(95% CI）** |  | **eGFR＜60 vs. eGFR≥90**  **OR(95% CI）** |
| **Simple renal cyst, yes vs. no** | 1.26*** (1.17-1.35) |  | 1.35 *** (1.16-1.56) |  |  |  |  |
| **Simple renal cyst number and size** |  |  |  |  |  |  |  |
| Number＜2 and size＜2cm vs. no |  |  |  |  | 1.29***(1.16-1.42) |  | 1.27* (1.03-1.57) |
| Number＜2 and size≥2cm vs. no |  |  |  |  | 1.19* (1.04-1.37) |  | 1.20 (0.91-1.57) |
| Number≥2 and size＜2cm vs. no |  |  |  |  | 1.34* (1.07-1.36) |  | 1.53 (0.99 -2.38) |
| Number≥2 and size≥2cm vs. no |  |  |  |  | 1.24* (1.06-1.46) |  | 1.68** (1.25-2.23) |
| **Age≥60 years vs.＜60 years** | 9.82***(9.27-10.40) |  | 11.14 *** (9.79-12.67) |  | 9.82***(9.27-10.44) |  | 11.12*** (9.77-12.65) |
| **Male vs. female** | 1.60*** (1.51-1.71) |  | 0.75 *** (0.66-0.85) |  | 1.61*** (1.51-1.71) |  | 0.75*** (0.66 -0.85) |
| **Hypertension, yes vs.no** | 1.55*** (1.47-1.65) |  | 1.75 *** (1.54-1.98) |  | 1.55*** (1.47-1.65) |  | 1.74*** (1.54-1.97) |
| **Diabetes mellitus，yes vs.no** | 0.85** (0.77-0.94) |  | 1.14 (0.94-1.37) |  | 0.85** (0.77-0.94) |  | 1.13 (0.94-1.36) |
| **Uric acid＞420umol/L vs.≤420umol/L** | 1.73*** (1.61-1.85) |  | 3.37 *** (2.93-3.88) |  | 1.73*** (1.61-1.85) |  | 3.38*** (2.93-3.89) |
| **Triglyceride＞1.7mmol/L vs.≤1.7mmol/L** | 1.06 (0.99-1.12) |  | 0.88 ** (0.78-0.99) |  | 1.06 (0.99-1.12) |  | 0.88 * (0.76-0.99) |

eGFR slight decline**:** 60≤eGFR＜90 ml/min per1.73m^2^, eGFR severe decline: eGFR＜60 ml/min per1.73m^2^

OR, odds ratio; CI: confidence interval; eGFR, estimated glomerular filtration rate calculated by the 2009 CKD-EPI formula.

**p*＜0.05, ***p*＜0.01, ****p*＜0.001
