# Supplementary figures and images for: Multiple and large simple renal cysts are associated with glomerular filtration rate decline: a cross-sectional study of Chinese population
Source: Eur J Med Res. 2024 Jan 3;29:11. doi: 10.1186/s40001-023-01552-2 (PMC10763358; doi:10.1186/s40001-023-01552-2)

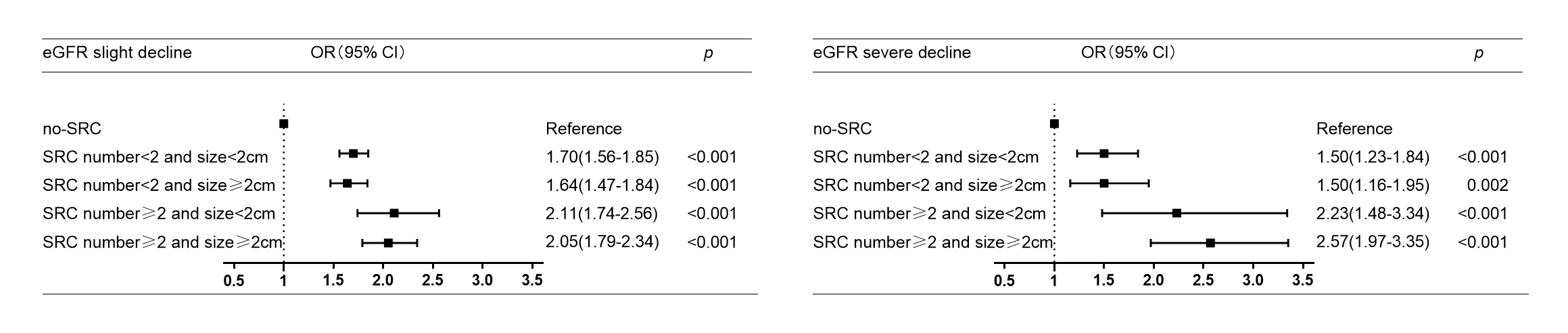

Supplement: Supplementary file 2 — Additional file 2: Figure S1. The unadjusted OR of SRC four combinates on the risk of eGFR decline. eGFR slight decline, 60 ≤ eGFR < 90 ml/min per1.73m2; eGFR severe decline, eGFR < 60 ml/min per1.73m2; OR odds ratio, CI confidence interval, eGFR estimated glomerular filtration rate calculated by the 2009 CKD-EPI formula, SRC simple renal cyst. [file 40001_2023_1552_MOESM2_ESM.tif]
